# Cheatomaly: weakly supervised video anomaly ranking for exam cheating detection using vision transformers

**DOI:** 10.3389/fdata.2026.1817120

**Published:** 2026-05-12

**Authors:** El Mehdi Alaoui Mrani, Anas Bouayad, Khalid Fardousse

**Affiliations:** 1 LIASSE Laboratory, ENSA, SMBA University, Fes, Morocco; 2 L3IA Laboratory, Faculty of Sciences, SMBA University, Fes, Morocco

**Keywords:** anomaly detection, cheating detection, weakly supervised learning, multiple instance learning, vision transformers, video analysis, temporal modeling

## Abstract

Detecting cheating in classroom examinations is challenging because suspicious behaviors are often subtle, temporally sparse, and context-dependent. To address the lack of dedicated benchmarks for this setting, we introduce Cheatomaly, a curated video dataset assembled from publicly available classroom examination material and annotated to support weakly supervised anomaly detection. We formulate cheating detection as a weakly supervised video anomaly ranking task using Multiple Instance Learning (MIL) with Vision Transformer features. Videos are divided into temporal segments, and segment-level representations are built using mean pooling and a Mean, Standard Deviation, and Temporal Difference (MSD) formulation. A margin-based ranking objective is used to prioritize anomalous videos and suspicious temporal segments using only video-level labels during training. Experimental results on Cheatomaly show strong video-level discrimination and meaningful frame-level localization across repeated runs. Ablation, baseline, statistical, and sensitivity analyses indicate that temporal aggregation affects the trade-off between ranking and localization but does not produce consistent statistically significant gains. Overall, Cheatomaly provides a realistic benchmark for studying subtle cheating-related anomalies in classroom examinations, and the results highlight that the main challenge lies in modeling context-dependent temporal behavior rather than feature aggregation alone.

## 1 Introduction

Anomaly detection using camera surveillance footage is a core problem in computer vision and video processing; the main challenge lies in the ambiguous and unpredictable nature of anomalies, which can differ depending on the moment, context, or location. As a consequence, ranking suspicious events can be more practical than binary detection, especially when precise temporal labels are unavailable.

In addition, public video anomaly datasets often cover a narrow range of scenarios. Many existing benchmarks focus on visually salient or well-defined abnormal events, which are easier to distinguish from normal activities. However, they do not adequately capture subtle and context-dependent behaviors such as those observed in classroom examination settings. As a result, these datasets provide limited insight into anomaly detection under ambiguous real-world conditions. Therefore, developing a dataset targeting such underexplored scenarios can help extend current evaluation settings and better reflect real-world challenges.

Cheating is one such issue that undermines fairness and trust within educational systems (Hu et al., 2024; Singh et al., 2024). It is a persistent challenge that negatively affects the quality and credibility of educational assessments. To address this concern, many institutions have installed classroom surveillance cameras as a complement to human supervision, aiming to strengthen academic integrity during examinations. However, manual monitoring is inherently limited by fatigue, subjectivity, and the difficulty of consistently identifying subtle suspicious behaviors across extended periods. In this context, recent advances in deep learning have made automated video-based analysis a promising approach for supporting anomaly detection in examination environments.

In this paper, we focus exclusively on real in-person examinations rather than online settings. We construct a benchmark from publicly available open-source video data relevant to classroom cheating scenarios, specifically organized for anomaly detection in exam environments. This work addresses cheating through a weakly supervised video anomaly ranking approach. Since cheating often manifests through irregular and context-dependent behaviors (Hussein et al., 2022), the model ranks deviations from normal activity through anomaly scoring rather than relying on strict binary labels. The dataset is annotated at the frame level to support detailed evaluation and temporal analysis.

The central problem addressed in this work is how to identify anomalous behaviors related to cheating from classroom video recordings in offline exams. Traditional methods rely heavily on manual human monitoring, which is prone to fatigue and subjectivity. The unpredictable nature of cheating—ranging from the use of hidden devices to subtle gestures—makes it challenging to define fixed behavior patterns. Based on these challenges, this work focuses on applying a weakly supervised anomaly ranking approach to subtle and context-dependent cheating behaviors, while analyzing the impact of realistic classroom conditions on detection performance. It further examines how dataset design and temporal representation influence the balance between anomaly ranking and temporal localization.

In this paper, we make the following contributions:

- We introduce Cheatomaly, a benchmark dataset for video anomaly detection in classroom examination settings, curated from publicly available sources and designed to capture subtle, context-dependent cheating behaviors.
- We formulate cheating detection as a weakly supervised video anomaly ranking problem, which avoids the need for precise temporal annotations and better reflects the ambiguity and sparsity of cheating events in real-world exam environments.
- We analyze temporal segment representations for anomaly ranking, including a Mean+Standard Deviation+Temporal Difference (MSD) formulation, and show that feature aggregation has limited impact on performance compared to the inherent complexity of the task.
- We provide a comprehensive evaluation protocol combining multiple data splits and repeated runs, with both video-level ranking and frame-level localization metrics, highlighting the challenges of fine-grained anomaly detection in realistic examination scenarios.

The structure of this paper is as follows. Section 2 reviews the existing work on video anomaly detection and cheating detection methods. Section 3 clarifies key definitions and concepts to properly position our study. Section 4 describes the Cheatomaly dataset, including its collection process, statistics, and data extraction pipeline. Section 5 presents the weakly supervised video anomaly ranking framework, together with the experimental setup and evaluation protocol. Section 6 reports and discusses the experimental results at both the video and frame levels.

## 2 Related work

Video anomaly detection (Chandola et al., 2009; Berroukham et al., 2023) has been extensively studied, particularly in surveillance contexts. Most approaches learn normal patterns and detect deviations at test time due to the rarity of anomalies. Early methods rely on unsupervised reconstruction or temporal prediction models, such as Autoencoders and ConvLSTM architectures (Hasan et al., 2016; Wang and Yang, 2022; Gong et al., 2019). However, these approaches often assume that abnormal events are sufficiently distinct from normal patterns, which limits their effectiveness in scenarios where anomalies are subtle and highly context-dependent.

Weakly supervised learning has been widely adopted in video anomaly detection (Hu et al., 2020) to address the scarcity of frame-level annotations. In particular, Multiple Instance Learning (MIL) enables training with video-level labels by learning to rank temporal segments based on anomaly scores. While this formulation reduces annotation effort and captures temporal structure, existing works are primarily evaluated on datasets where anomalous events remain relatively distinguishable from normal behavior.

Cheating detection has been explored in both online and in-person examination contexts. Classroom-based approaches often rely on predefined actions such as object usage or head movements, while online systems focus on gaze tracking, facial cues, or desktop monitoring (Ramzan et al., 2024). These methods typically depend on supervised learning and explicitly defined behavioral patterns, which limits their ability to capture subtle, adaptive, or previously unseen cheating strategies.

Most publicly available video anomaly datasets focus on generic surveillance scenarios such as violence, theft, or traffic incidents. These anomalies are typically visually salient and easier to distinguish from normal behavior. In contrast, datasets specifically targeting cheating detection are often limited, domain-specific, or oriented toward object detection tasks. As a result, they do not adequately capture the subtle and context-dependent nature of cheating behaviors in real classroom examination environments. This gap motivates the need for datasets that better reflect ambiguous real-world conditions.

Many existing approaches rely on supervised learning, making detection dependent on predefined behavioral patterns. Conversely, reconstruction-based unsupervised models assume a clear distinction between normal and abnormal events, which may not hold in context-dependent scenarios (Murali et al., 2025). In settings such as classroom examinations, where anomalies are subtle and ambiguous, these assumptions become limiting. This highlights the need for approaches that can model deviations without relying on strict definitions of abnormal behavior.

## 3 Key definitions and problem formulation

### 3.1 Anomaly detection

Anomaly detection (Chandola et al., 2009) in videos refers to identifying patterns or behaviors that deviate from what is expected or considered normal, including unusual activities, objects, people, or events that do not conform to the situation. The anomaly detection can use various techniques or models, those methods range from classical computer vision techniques to advanced machine learning, deep learning, and hybrid models (Aslam and Kolekar, 2024). Most video anomaly detection approaches assume access to normal data during training and learn representations of expected patterns. During testing, deviations from this learned normality are treated as anomalies, through anomaly scores or threshold-based decisions. However, this formulation becomes challenging with subtle, ambiguous, or highly context-dependent anomalies (Berroukham et al., 2023; Liu et al., 2023). The general purpose is to detect anomalies automatically, in a significant way and in a reasonable amount of time (Singh et al., 2020) to get the opportunity to move accordingly and take the right decision, especially in some situations where the anomaly passes quickly or requires urgent interference.

### 3.2 Anomaly ranking

The nature of anomalies in real-world scenarios makes it hard to establish clear boundaries between normal and abnormal events. To address this problem, we adopt an anomaly ranking formulation (Sultani et al., 2018); each temporal segment of a video receives a score reflecting its abnormality, allowing suspicious segments to be prioritized rather than strictly labeled. Ranking is more suitable when frame-level annotations are unavailable, and it is more flexible with ambiguous or context-dependent anomalies. The model focuses on relative abnormality instead of strict categorization.

### 3.3 Weakly supervised Video Learning

Fully supervised anomaly detection is considered a frame-level classification problem, requiring detailed temporal annotations, while such annotations are costly and often impractical in real-world scenarios due to the nature of anomalies. This motivates weakly supervised learning, as only video-level labels are usually available in realistic scenarios. It offers a practical compromise between supervised classification and unsupervised reconstruction-based detection. Weakly supervised approaches are commonly implemented using Multiple Instance Learning (Sultani et al., 2018); in MIL each video is treated as a bag, temporal segments are treated as instances, and only the bag label is known. Positive bags have at least one anomalous segment, and negative bags have only normal segments. The model therefore learns to assign higher anomaly scores to segments from positive bags compared to segments from negative bags. And based on this score, we rank temporal segments with the objective to rank anomalous segments higher than normal ones. This aligns well with cheating behavior and reflects real monitoring scenarios.

### 3.4 Cheating in classroom examination settings

Cheating remains a persistent concern in universities and schools; it remains a persistent challenge that affects the fairness and reliability of the evaluation (Javed et al., 2023). Although examination supervision traditionally relies on human proctors, many institutions now complement human monitoring with classroom surveillance cameras (Khel et al., 2023; Vally et al., 2024). However, supervising multiple students simultaneously remains cognitively demanding and manual observation is limited by human capabilities. This motivates the exploration of automated video-based analysis for these supervision processes.

In classroom settings, cheating behaviors can generally be grouped into two categories: interaction-based cheating, where cheating involves communication or coordination between students, such as whispering, passing papers, or hand signals. This type of cheating requires spatial relationships modeling and temporal synchronization between students. The second category is solo cheating, where cheating is performed independently by a single student. Mostly it involves hidden notes, mobile phones, and subtle body movements (Tran et al., 2023). It is characterized by small, localized behavioral deviations. Both categories involve behavioral deviations rather than extreme actions. These behaviors are typically spatio-temporal anomalies, such as unusual objects and sudden or repeated suspicious movements. These anomalies occur in classrooms that are sparsely to moderately crowded. Those characteristics make clear binary boundaries difficult to define and the cheating a structured anomaly ranking problem rather than a simple classification task.

In our case, cheating is not treated as a predefined action class; we do not rely on fixed behavioral templates. Instead, we model it as a deviation within a structured classroom context. The classroom is a structured environment characterized by recurring normal behavioral patterns, and the deviations are subtle, context-dependent, and temporally localized (Doshi and Yilmaz, 2022). We adopt a weakly supervised anomaly ranking method, aiming to prioritize suspicious temporal segments rather than imposing a strict binary classification. This formulation reflects realistic monitoring scenarios where automated scoring supports, rather than replaces, human judgment.

## 4 Dataset description

**Figure 1:**
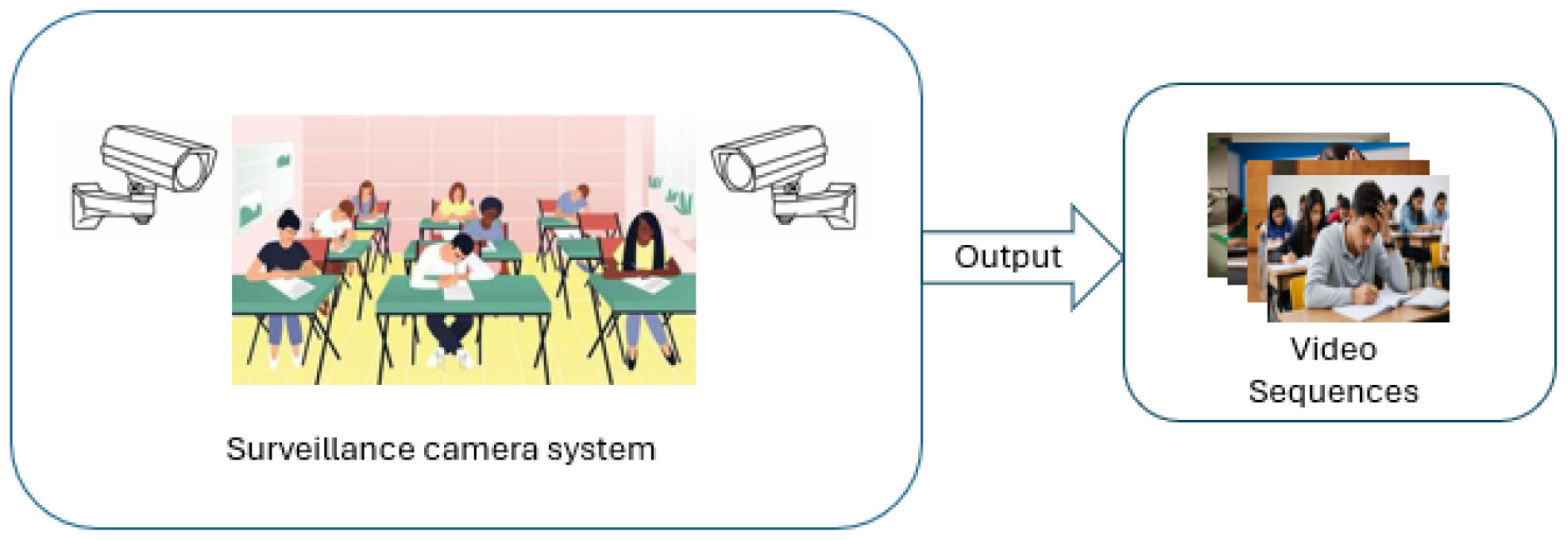
Data collection.

The cheatomaly dataset is composed of real in-person classroom examination sessions. It captures students' behavior under standard exam conditions, in both normal activities and cheating situations (Figure 1). Rather than focusing on object detection only, our dataset explores subtle, temporally localized behavior patterns that are context-dependent. This structure allows modeling subtle, context-dependent behaviors, ensuring that the content captures and highlights unusual patterns or events. This involves a detailed process of curating, structuring, and preprocessing publicly available data to support effective training and anomaly analysis.

### 4.1 Dataset extraction

**Figure 2:**
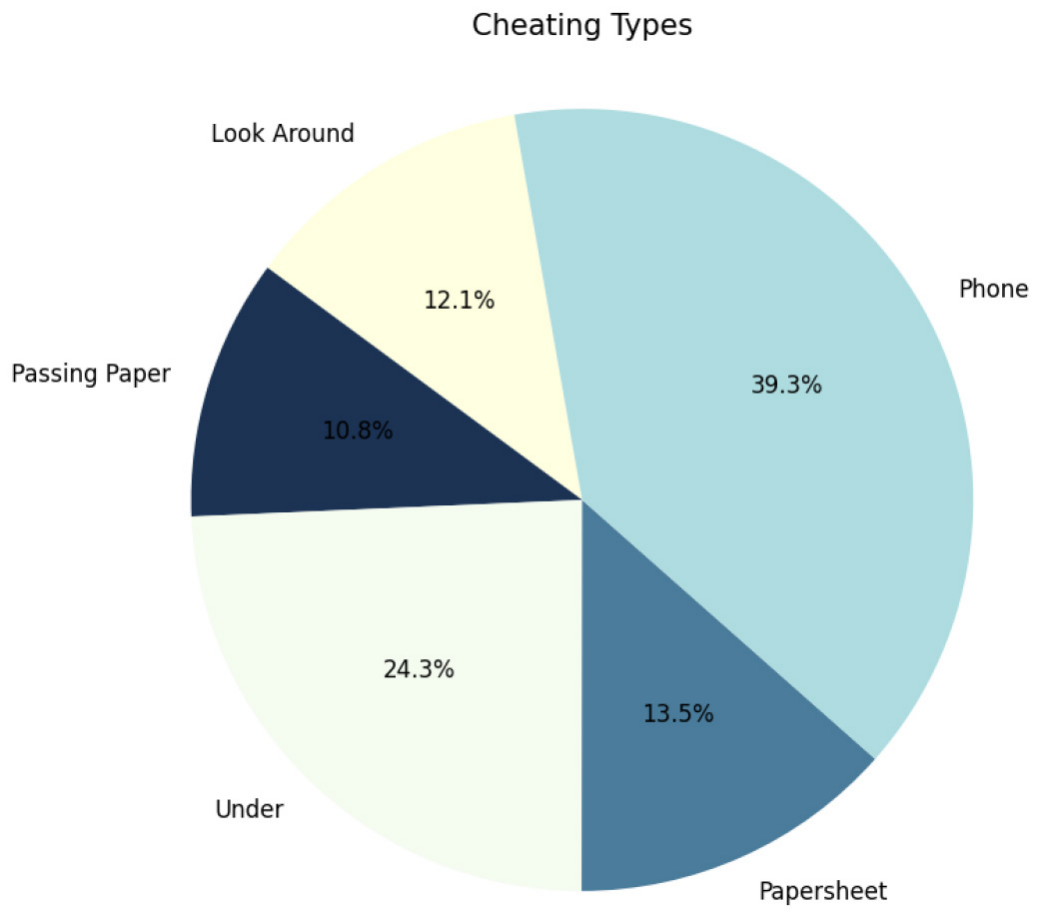
Distribution of specific cheating anomalies within the dataset.

The dataset consists of 94 video sequences gathered from publicly available online sources and open datasets. In this work, only the video content was retained from the original sources, and the most relevant clips were carefully selected and organized to reflect examination-related situations. The selected videos were then structured to include both normal and anomalous student behaviors in realistic assessment-like settings. Since the original sources were diverse and did not always provide annotations suited to the objective of this study, the labeling was carried out manually by the authors for all selected videos. This process made it possible to build a coherent and task-oriented dataset for training and anomaly analysis while preserving diversity in viewpoints, scene configurations, and behavioral patterns. The diversity is ensured by considering different types of cheating (see Figure 2), categorized into two general types:

- Solo cheating: the cases where a student is cheating by himself, using some cheating tools, such as small sheets or pieces of paper already prepared to use in the exams, cell phones as well, or other gadgets like headphones, the VIP card, and other commonly reported concealment strategies.
- Interactive cheating: the cases where two or more students are helping each other or passing answers, the anomaly can be talking to each other, passing papers, or even making signs that can be interpreted as answers in exams or gesture-based signaling for the correct answer.

### 4.2 Dataset labeling

The dataset is designed to support anomaly detection analysis. During training, only video-level labels are used, and frame-level annotations are used exclusively for evaluation. This setup reflects realistic annotation constraints in surveillance scenarios.

**Figure 3:**
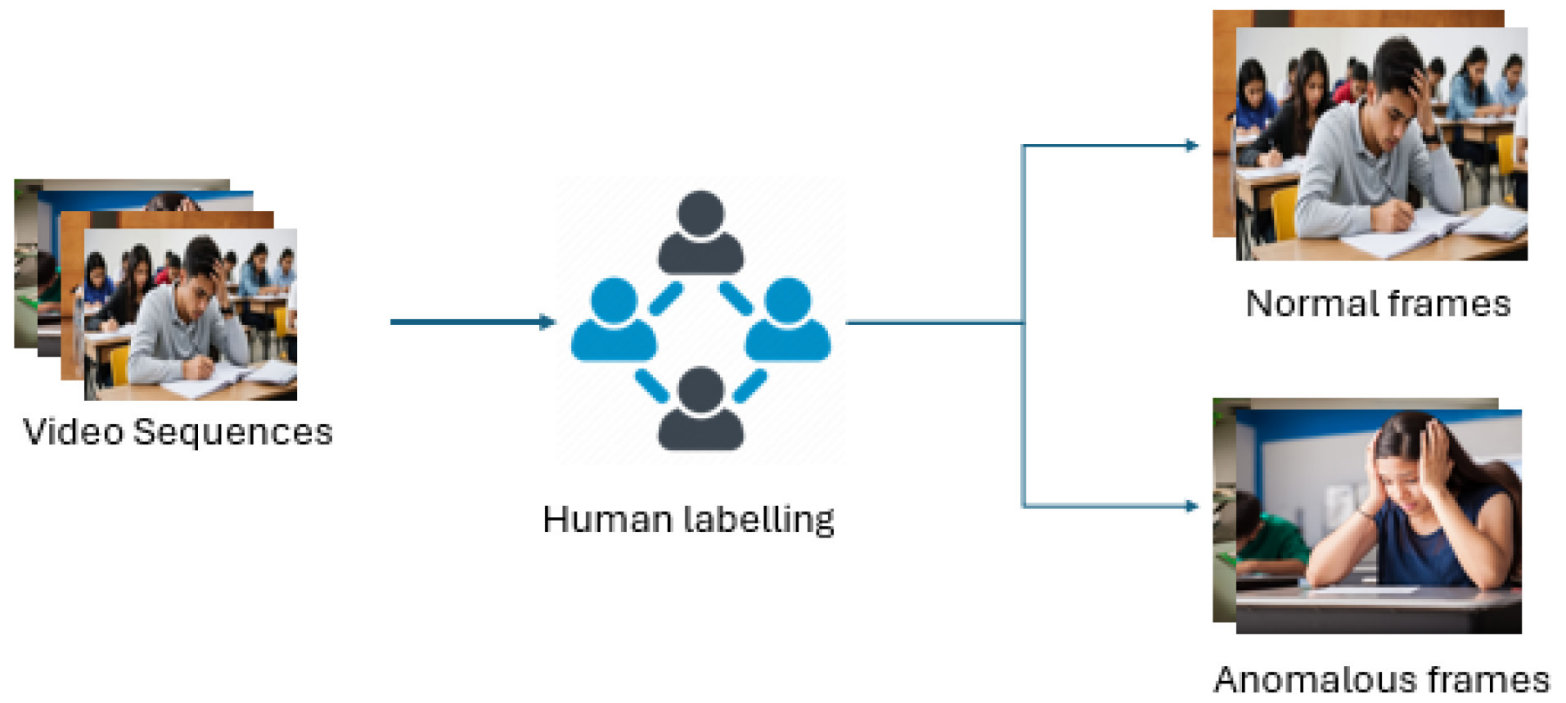
Data labeling.

The testing set was manually annotated by two independent reviewers (Figure 3). The annotation process was carried out by two of the authors independently. Each annotator reviewed the entire set of videos separately, marking the temporal intervals corresponding to cheating behavior. After completing their individual reviews, both annotators compared their results, discussed differences, and confirmed the final anomalous intervals by mutual agreement.

Cheating cannot be inferred from a single frame; only temporally coherent segments were labeled anomalous. Annotations were made while considering both spatial context and temporal continuity. Consequently, entire intervals corresponding to cheating behavior were marked. Overall, the testing dataset includes 16,561 normal frames and 19,464 anomalous frames distributed across 46 video sequences summarized in Table 1.

**Table 1:** Summary statistics of the Cheatomaly dataset.

Dataset property	Value
Total video sequences	94
Video duration	30
Resolution	1,920 × 1,080
Frame rate	30 FPS
Experimental split	
Training videos (normal only)	48
Testing videos (mixed)	46
Total test frames (normal)	11,918
Total test frames (anomalous)	15,551
Cheating type distribution	
Mobile phone usage	51.4%
Under-table material	24.3%
Cheat sheet (paper)	13.5%
Passing papers/interaction	10.8%

These labels serve as ground truth during evaluation, allowing us to assess the ranking of anomalous segments with metrics such as PR-AUC and ROC-AUC. Evaluation is performed at the video level (ranking performance) and frame level (temporal localization performance); These annotations enable quantitative evaluation of ranking and localization performance.

The video material used in this study was gathered from publicly available online sources and open datasets. In order to preserve behavioral cues relevant to the task, such as head movement and posture, the original visual content was retained during annotation and analysis. The collected material was used solely for research purposes and handled with due care in line with principles of responsible data use.

### 4.3 Challenges and limitations

Constructing a real-world video dataset involves multiple practical constraints. Moreover, making it support anomaly detection analysis in exams introduces additional complexity. The classroom exam environments contain natural variability. In particular, camera position, angle, and lighting conditions vary across recordings. As a result, parts of hands, desks, or objects frequently appear. Consequently, the cheating behavior became harder to localize and interpret. These characteristics increase the difficulty of reliable anomaly modeling.

Anomaly detection models flag behaviors that deviate from the learned normal patterns. This characteristic has two implications. On the one hand, previously unseen or emerging forms of cheating can be detected without explicit supervision. On the other hand, certain unusual but non-cheating behaviors may also be assigned high anomaly scores, resulting in false positives. While this may slightly affect precision, it does not contradict the objective of highlighting suspicious events for further review. A ranking-based formulation partially alleviates this issue by prioritizing the most suspicious temporal segments instead of enforcing rigid binary decisions. Nevertheless, the balance between sensitivity and precision remains an inherent trade-off in anomaly-based approaches.

The dataset also presents additional challenges, such as the significant difference between solo and interactive cheating. Solo cheating relies on subtle individual cues, while interactive cheating depends on coordinated multi-person interactions; these differences introduce distinct modeling challenges.

To address these limitations, we intend to expand this dataset to include different classrooms, lighting conditions, and cheating situations and tools, enhancing robustness and diversity for broader research use.

## 5 Methodology and problem formulation

### 5.1 Framework overview

In this work, we formulate cheating as a weakly supervised video anomaly ranking problem, rather than a strict classification task. We aim to identify and prioritize suspicious temporal segments within a video sequence instead of assigning a single normal or abnormal label to it. The framework operates under weak supervision, meaning that only video-level labels are available. During training, we aim to encourage higher anomaly scores for segments originating from positive videos compared to those from negative videos. Each video is first decomposed into short temporal segments. Visual features are extracted from frames using a deep backbone network and aggregated into segment-level representations. These segments are treated as instances within a Multiple Instance Learning (MIL) setting, where the entire video corresponds to a bag (Sultani et al., 2018). The model assigns an anomaly score to each temporal segment, reflecting its relative abnormality within the video. This ranking-based formulation allows the model to learn discriminative patterns without requiring precise temporal annotations. At inference time, segment-level scores are used both to compute a global video anomaly score and to localize suspicious intervals within the sequence.

### 5.2 Problem formulation

#### 5.2.1 From video to temporal instances

In our setting, to capture temporal dynamics, each classroom examination video is considered a temporal sequence and not a static sample. A video V of length T frames is divided into overlapping temporal segments (Sultani et al., 2018) to capture short-term behavioral patterns. Each segment presents a contiguous window of frames, and it is represented by a feature vector extracted using a pre-trained visual backbone.

This segmentation step is important because cheating anomalies are rarely global events and they don't affect entire videos. They are localized temporal deviations embedded within normal behavior (Tang et al., 2020; Naji et al., 2023). Therefore, instead of assigning a label to each frame during training, we represent each video as a collection of temporal instances. A video is partitioned into N temporal segments, producing a set of feature vectors, each vector encodes the visual representation of a short temporal window. The set is treated as a bag in a Multiple Instance Learning framework (Carbonneau et al., 2018), and only the video level label is available in training, indicating if the video contains cheating (1) or not (0).

#### 5.2.2 Feature extraction with vision transformer (ViT)

Each temporal segment must be represented by a compact and discriminative feature vector. Training deep spatial models from scratch requires large annotated datasets, which are not available in our setting. We rely on a pretrained visual backbone. Therefore, we use a pre-trained Vision Transformer (ViT) as the spatial feature extractor (Dosovitskiy et al., 2021).

Vision Transformers use self-attention mechanisms (Vaswani et al., 2017) that allow modeling long-range dependencies between different regions of the images. This is particularly useful in classroom scenes where interactions matter, whether between hands, desks, objects, or students.

For each frame within a temporal segment, the ViT backbone produces a fixed-dimensional embedding vector that captures high-level spatial information. We use the final representation produced by the network, corresponding to the global token (CLS) or an equivalent pooled embedding, as the descriptor of the frame. We keep the backbone frozen during training and only optimize the MIL ranking module to avoid overfitting and preserve the robustness of the learned features. This process results in a sequence of frame-level embeddings for each temporal window, which form the basis for segment-level aggregation and anomaly scoring.

To represent each temporal segment, frame-level embeddings are aggregated using statistical pooling. Cheating behaviors in classroom exams are often subtle, localized, and temporally short; therefore, simple mean pooling may smooth out subtle temporal variations. To capture intra-segment variability and motion dynamics, we incorporate additional statistics such as standard deviation and temporal differences between consecutive frames. This representation incorporates additional temporal variability and motion dynamics, allowing us to analyze its impact on anomaly ranking and localization (Ullah et al., 2022; Alaoui Mrani et al., 2024). Each resulting instance representation is treated as an instance within a Multiple Instance Learning (MIL) framework, where a full video is modeled as a bag of temporal segments and only the video-level label is available during training (Sultani et al., 2018). Frame-level annotations are used exclusively for evaluation. This feature extraction strategy bridges pre-trained visual representations with weakly supervised anomaly ranking, enabling the modeling of subtle cheating behaviors without requiring dense temporal supervision.

### 5.3 Anomaly scoring and ranking objective

After extracting segment-level feature vectors, each temporal segment must be assigned an anomaly score. This score reflects the relative abnormality of the segment. Therefore, higher scores indicate a stronger deviation from normal classroom behavior.

To handle weak supervision, the training setup follows the Multiple Instance Learning framework. Each video is treated as a bag of temporal segments. During training, only the video-level label is available. A positive video contains at least one cheating segment and a negative video contains only normal behavior. Since frame-level labels are not used during training, the model must infer which segments inside a positive video are responsible for the anomaly.

We aggregate individual segment scores to generate a final video-level prediction. Depending on the specific configuration, the system selects either the single highest-scoring segment or the Top-K segments. This reflects the fundamental MIL assumption that a positive video contains at least one anomalous segment. This follows the MIL assumption that positive videos contain at least one anomalous segment. The peak anomaly score effectively becomes the proxy for the entire video.

The proposed ranking objective operates at the bag level while relying on latent instance-level scores. Specifically, segment-level features are first mapped to anomaly scores, which are then aggregated to produce a single score per video. This aggregation step reflects the Multiple Instance Learning (MIL) assumption: in the standard setting, max pooling approximates the presence of at least one anomalous segment, whereas Top-K aggregation relaxes this assumption by considering multiple high-scoring segments. The ranking loss then enforces that anomalous videos obtain higher aggregated scores than normal ones by a margin, without requiring explicit temporal annotations. This formulation allows the model to focus on the most informative segments while remaining robust to noise and temporal ambiguity in real classroom scenarios.

Our learning objective is based directly on this aggregation. We optimize the model to encourage the highest-scoring segment within a positive (cheating) video score strictly higher than any routine segment in a negative (normal) video. Applying a margin constraint enforces a clear mathematical separation between the two classes. We are not attempting to classify every frame. Instead, the framework encourages anomalous segments to rank above normal classroom behavior.

Real-world cheating is often subtle and highly localized in time. The exact boundary where normal behavior transforms into an academic violation is often ambiguous. Although a strict binary classifier requires precise temporal annotations to function, our ranking approach handles this inherent uncertainty much more flexibly. It prioritizes highly suspicious segments without forcing the system to make hard binary decisions. The resulting anomaly scores are designed to flag footage for human review, not to autonomously accuse a student.

Overall, this ranking-based learning strategy aligns naturally with the weak supervision setting. It enables detection of subtle cheating behaviors without requiring dense frame-level labels. At the same time, it provides interpretable anomaly scores for both video-level and temporal evaluation.

### 5.4 Training strategy

The model is trained under a weakly supervised multiple instance learning setting. During training, each iteration samples one anomalous bag and one normal bag. A bag corresponds to a video represented as a set of temporal segment feature vectors.

The ranking network is optimized using the Adam optimizer with a learning rate of 1e-4. The Vision Transformer backbone remains frozen throughout training, and only the parameters of the ranking network are updated. This design choice stabilizes training and reduces the risk of overfitting given the limited dataset size.

Training is performed for 10 epochs, with 300 steps per epoch. At each step, the model receives one positive bag (containing at least one anomalous segment) and one negative bag (containing only normal segments).

Each segment receives a scalar anomaly score from a lightweight MLP. Instead of averaging all segments, segment scores are aggregated using Top-K pooling. Specifically, the highest 10% of segment scores within a bag are averaged (TOPK_FRAC = 0.10). This mechanism prevents isolated noisy peaks from dominating the bag score and focuses learning on the most suspicious temporal regions.

The ranking objective follows a margin-based formulation. The loss enforces that the anomalous bag score exceeds the normal bag score by a margin of 1.0 (MARGIN = 1.0). Additionally, a small L2 regularization term is applied to segment scores to stabilize training. In order to avoid leakage between related videos (split recordings of the same exam), a group-based split protocol is applied. Videos sharing the same base identifier are kept within the same partition. Sixty percent of groups are used for MIL training, and the remaining 40 percent for evaluation (GROUP_SPLIT_FRAC = 0.60, RANDOM_SEED = 42).

We prioritized stability and consistent ranking over aggressive optimization when tuning our hyperparameters. Cheating is a temporally sparse event; it usually only happens during a tiny fraction of a recorded exam. To capture this reality, we applied Top-K pooling at a 10% threshold. Relying on a single maximum score makes the system far too sensitive to random visual noise, but averaging every segment simply washes out the brief moments of actual cheating. The same Top-K strategy is used consistently during both training and evaluation. The 10% mark provided a practical balance between sensitivity and robustness.

We also set the margin constraint to 1.0. This encourages a clear separation between positive and negative video bags without risking unstable gradients during training. Finally, we kept the Vision Transformer (ViT) backbone entirely frozen. Given our weakly supervised setup and the limited size of the dataset, fine-tuning the ViT could increase the risk of overfitting. Overall, these configuration choices aim to ensure the ranking dynamics remain tightly controlled and the final results are fully reproducible.

To ensure robustness and reduce sensitivity to random initialization, each experiment is repeated across 15 different random seeds. Reported results correspond to the mean and standard deviation computed over all runs.

### 5.5 Evaluation protocol

Evaluation is conducted at both the video level and the frame level.

#### 5.5.1 Video-level evaluation

For each evaluation video, the model computes segment-level anomaly scores. These scores are aggregated using the same Top-K strategy used in training. The results represent the video-level anomaly score. To measure the model's performance, we used Precision-Recall Area Under Curve (PR-AUC) and Receiver Operating Characteristic Area Under Curve (ROC-AUC) (Fawcett, 2006). These metrics evaluate the model's ability to assign higher scores to cheating videos than to normal videos.

#### 5.5.2 Frame-level evaluation

Although we use a weakly supervised model, with training using only video-level labels, frame-level annotations are available for testing. To assess temporal localization performance, we map back segment scores to frame indices; each frame inherits the anomaly score of its corresponding segment, and we compare the scores to the ground-truth frame annotations. Frame-level PR-AUC and ROC-AUC quantify the model's ability to localize cheating behavior over time.

#### 5.5.3 Feature modes comparison

Two feature representations are evaluated: mean pooling and Mean + Standard Deviation + Temporal Difference (MSD). Both configurations are trained independently under the same protocol to analyze the impact of temporal aggregation strategies.

The main training and reproducibility settings used in all experiments are summarized in Table 2.

**Table 2:** Training and reproducibility configuration of the proposed weakly supervised anomaly ranking framework.

Component	Configuration
Optimizer	Adam
Learning rate	1 × 10^−4^
Margin (ranking loss)	1.0
Top-k fraction	10%
Epochs	10
Steps per epoch	300
Visual backbone	ViT-Base (google/vit-base-patch16-224-in21k)
Backbone training	Frozen
Random seed	42

#### 5.5.4 Limitations

While the proposed framework demonstrates promising results, we acknowledge certain limitations of the current study. First, the Cheatomaly dataset remains relatively small, which may limit the generalization of the learned patterns. Second, the evaluation focuses on controlled ablation experiments rather than direct comparison with existing state-of-the-art methods, as our primary objective is to isolate and assess the contribution of the proposed segment representation. Finally, although a sensitivity analysis of key hyperparameters is included in the Results and Discussion section, further extensive evaluation across broader settings remains an important direction for future work.

## 6 Results and discussion

The main objective of this study is to analyze how a weakly supervised anomaly ranking framework behaves in realistic examination environments. The evaluation is conducted at both the video level and the frame level in order to assess ranking performance and temporal localization capability. All results are averaged over five group-based splits and 15 random seeds, and are reported as mean ± standard deviation to ensure robustness against both data partitioning and random initialization variability.

### 6.1 Comparison with MIL baseline

To evaluate the proposed approach against a standard weakly supervised alternative, we compare different aggregation strategies within the same Multiple Instance Learning (MIL) ranking framework. In particular, we consider:

- MIL-Max baseline: standard max aggregation, where the bag score is defined as the maximum segment score, corresponding to the classical MIL assumption that a positive bag contains at least one anomalous instance.
- Top-K aggregation: the proposed strategy, where the bag score is computed as the average of the top-*K* highest segment scores.
- MSD representation: an enriched temporal feature representation combined with Top-K aggregation.

All methods share the same Vision Transformer backbone, ranking loss, training protocol, and evaluation setup. This controlled design ensures that differences are solely attributable to the aggregation strategy and feature representation.

As shown in Table 3, the MIL-Max baseline already achieves strong performance, particularly at the video level. This indicates that distinguishing anomalous from normal videos is relatively straightforward under weak supervision.

**Table 3:** Comparison of aggregation strategies under the same MIL ranking framework.

Method	Video PR-AUC	Frame PR-AUC
MIL-Max (baseline)	0.982 ± 0.011	0.709 ± 0.081
Mean + Top-K	0.9564 ± 0.0332	0.7809 ± 0.0922
MSD + Top-K	0.9498 ± 0.0398	**0.7843 ± 0.0927**

Results are reported as mean ± standard deviation over multiple runs. Bold values indicate the best performance for each metric.

However, frame-level performance remains significantly lower and exhibits noticeable variability across splits. This highlights the intrinsic difficulty of precise temporal localization in real classroom environments, where cheating behaviors are often subtle, sparse, and context-dependent.

Compared to MIL-Max, Top-K aggregation yields comparable video-level performance while providing a more stable estimate of anomaly scores by leveraging multiple high-scoring segments rather than relying on a single peak response. The MSD representation further refines temporal characterization, leading to marginal differences in frame-level performance.

Overall, these results suggest that the primary performance gains stem from the ranking formulation itself, while aggregation strategies mainly influence the trade-off between robustness and temporal localization rather than producing consistent improvements in global detection performance.

### 6.2 Video-level ranking performance

At the video level, the goal is to correctly rank examination videos according to the presence of cheating behavior. Using mean pooling, the model achieves a Video PR-AUC of 0.9564 ± 0.0332 and a Video ROC-AUC of 0.8345 ± 0.1330. Using the MSD representation (Mean + Standard Deviation + Temporal Difference), the model obtains a Video PR-AUC of 0.9498 ± 0.0398 and a Video ROC-AUC of 0.8098 ± 0.1167.

As shown in Table 3, both feature representations achieve comparable performance across all evaluation metrics. Mean pooling slightly outperforms the MSD representation at the video level, while MSD provides marginal differences in other metrics. Overall, the variations remain limited, indicating that global ranking performance is largely insensitive to the choice of feature aggregation.

These results indicate that the proposed ranking framework reliably prioritizes cheating videos over normal ones under weak supervision. Given the moderate class imbalance and the ranking nature of the task, PR-AUC is considered the primary metric, as it reflects how effectively the model prioritizes cheating videos. ROC-AUC is reported as a complementary measure of class separability.

**Figure 4:**
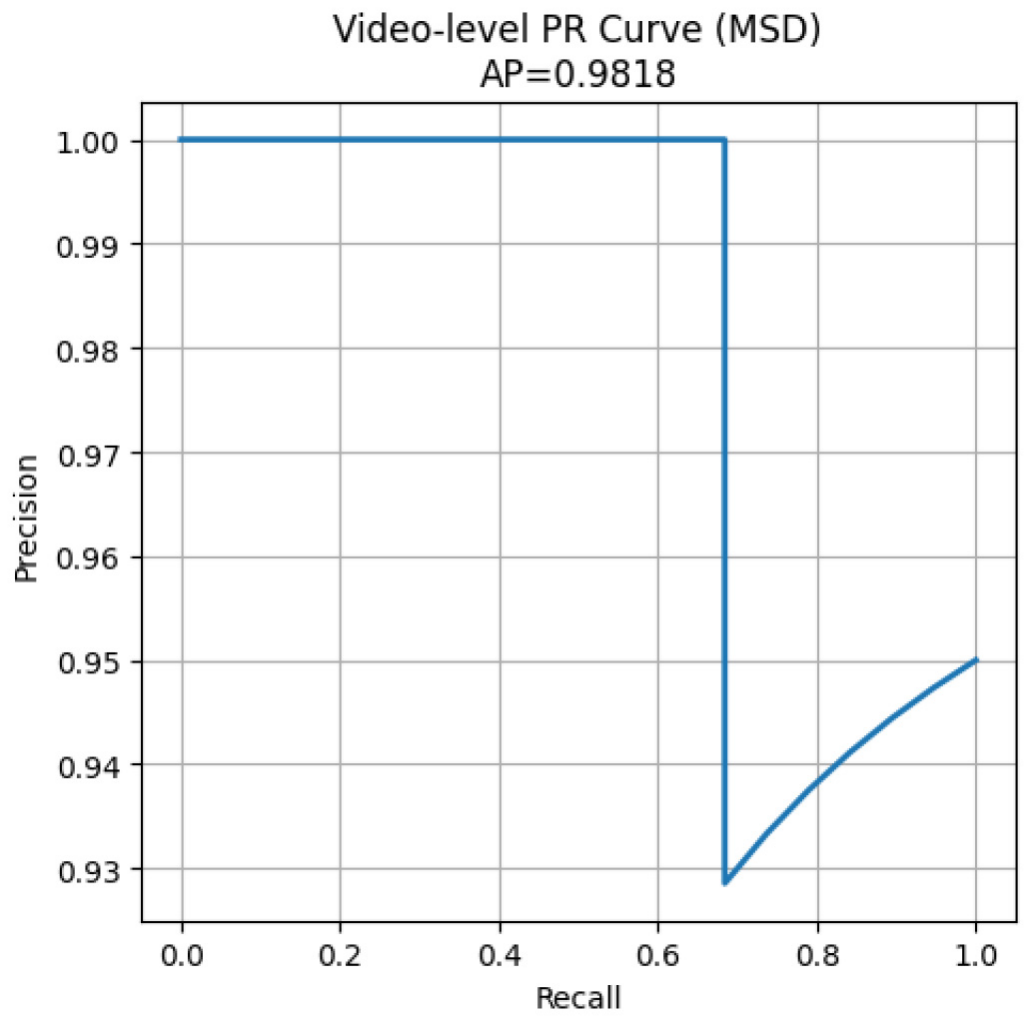
Video-level Precision–Recall curve for a representative evaluation run. The curve shows that the model maintains high precision across a wide range of recall values, indicating effective prioritization of cheating videos over normal ones under weak supervision.

To further illustrate the ranking behavior of the proposed method, Figure 4 presents the Precision–Recall curve for a representative evaluation run. The curve shows that the model maintains high precision across a wide range of recall values, confirming its ability to effectively prioritize anomalous (cheating) videos over normal ones.

### 6.3 Frame-level temporal localization performance

Although the model is trained using only video-level labels, frame-level annotations are available for evaluation. This allows assessing whether high anomaly scores correspond to actual cheating intervals.

Using mean pooling, the model achieves a Frame PR-AUC of 0.7809 ± 0.0922 and a Frame ROC-AUC of 0.7536 ± 0.0763. The MSD representation yields a Frame PR-AUC of 0.7843 ± 0.0927 and a Frame ROC-AUC of 0.7426 ± 0.0842.

These results confirm that both feature representations provide comparable temporal localization performance. The observed differences remain small and inconsistent across runs, indicating that segment-level aggregation alone is insufficient to substantially improve fine-grained temporal detection.

This gap is expected, as cheating behaviors are often subtle, short-lived, and context-dependent. In addition, segment-based scoring introduces temporal smoothing, which can attenuate abrupt anomalies when mapped back to individual frames.

**Figure 5:**
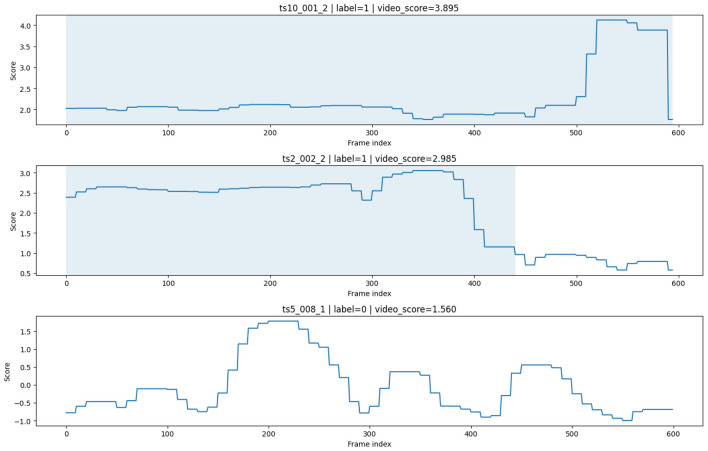
Frame-level anomaly score evolution for representative sequences. Peaks in anomaly scores generally align with annotated cheating intervals, indicating that the model captures relevant temporal patterns. However, fluctuations in normal segments highlight the presence of false positives and the ambiguity of subtle behaviors in examination environments.

Figure 5 illustrates frame-level anomaly score evolution for representative sequences. In anomalous videos, score peaks generally align with annotated cheating intervals, indicating that the model captures relevant temporal patterns. However, fluctuations are also observed in normal sequences, reflecting false positives and highlighting the ambiguity of subtle behaviors in exam environments.

### 6.4 Statistical analysis

To assess whether the observed differences between feature representations are statistically significant, we conduct paired *t*-tests across the 15 runs for each evaluation metric.

As shown in Table 4, none of the observed differences between Mean pooling and MSD representations are statistically significant (*p*>0.05). While each method shows slight advantages on specific metrics, these differences remain within the variability observed across runs.

**Table 4:** Statistical significance analysis between mean pooling and MSD representation across 15 runs.

Metric	Mean difference	*p*-value	Significance (*p* < 0.05)
Video PR-AUC	−0.0066	0.450	No
Video ROC-AUC	−0.0247	0.441	No
Frame PR-AUC	+0.0033	0.825	No
Frame ROC-AUC	−0.0110	0.380	No

In addition, the standard deviation values reported in Table 3 show comparable variability for both methods, and their performance ranges substantially overlap. This indicates that the observed differences are not consistently reproducible across different splits.

Overall, these results suggest that performance variations are primarily driven by run-to-run variability rather than the choice of feature representation. While MSD introduces additional temporal information, it does not yield statistically reliable improvements in this setting.

### 6.5 Sensitivity analysis

To assess the robustness of the proposed framework, we analyze the impact of key hyperparameters on performance, focusing on the Top-K selection ratio and the margin used in the ranking loss, as shown in Table 5.

**Table 5:** Sensitivity analysis of the MSD representation with respect to Top-k fraction and ranking margin.

Top-k	Margin	Video PR-AUC	Video ROC-AUC	Frame PR-AUC	Frame ROC-AUC
0.05	0.1	0.894	0.656	0.641	0.660
0.05	0.5	0.929	0.766	0.746	0.744
0.05	1.0	0.979	0.922	0.723	0.773
0.10	0.1	0.841	0.719	0.753	0.792
0.10	0.5	0.937	0.797	0.679	0.717
0.10	1.0	0.918	0.766	0.644	0.699
0.20	0.1	0.875	0.625	0.669	0.565
0.20	0.5	0.903	0.734	0.757	0.746
0.20	0.1	0.927	0.750	0.729	0.700
0.30	0.1	0.915	0.797	0.790	0.790
0.30	0.5	0.944	0.828	0.771	0.790
0.30	1.0	0.924	0.797	0.814	0.781

#### 6.5.1 Effect of Top-K ratio

The Top-K mechanism selects the highest-scoring temporal segments within each video to compute the final anomaly score. We evaluate different values of the Top-K fraction, ranging from small values (focusing on a few highly discriminative segments) to larger values (capturing broader temporal context). Results show that very small Top-K values tend to improve frame-level localization by focusing on peak anomaly segments, but may slightly reduce video-level stability. In contrast, larger Top-K values produce more stable video-level ranking performance but dilute the contribution of short and subtle anomalies. Overall, intermediate values provide a balanced trade-off between ranking accuracy and temporal localization.

#### 6.5.2 Effect of margin parameter

The margin parameter in the ranking loss controls the separation between normal and anomalous bags. We evaluate multiple margin values to assess its influence on convergence and performance. We observe that small margin values lead to weaker separation between classes, while excessively large margins can introduce instability during training. Moderate margin values provide the most consistent performance across runs, indicating that the framework is relatively stable within a reasonable parameter range.

#### 6.5.3 Discussion

Overall, the results indicate that the proposed framework is robust to moderate variations in key hyperparameters. While these parameters influence the balance between ranking and localization performance, no single configuration consistently dominates across all metrics. This behavior is consistent with the inherently subtle and context-dependent nature of cheating anomalies, where multiple temporal patterns may contribute to detection performance.

### 6.6 Qualitative and failure analysis

As summarized in Table 6, we analyze the qualitative behavior of the model to better understand its decision patterns and limitations in realistic examination settings.

**Table 6:** Failure cases observed in the Cheatomaly dataset and corresponding limitations.

Scenario	Observed limitation
Subtle cheating gestures	Weak anomaly scores due to minimal motion deviation
Occlusions (hands, desks)	Partial visibility reduces detection reliability
Ambiguous behaviors	Overlap between normal and suspicious actions
Temporal sparsity	Short anomalies diluted within long normal sequences
Environmental variability	Lighting and camera angle affect consistency

#### 6.6.1 False positives

The model frequently assigns high anomaly scores to normal behaviors that visually resemble cheating actions. These include repeated head movements, posture adjustments, or interactions with objects such as pens, papers, or desks. In multi-camera classroom settings, such behaviors can appear ambiguous due to viewpoint variations and partial occlusions. As a result, normal actions with noticeable motion or directional gaze are sometimes misclassified as anomalous.

#### 6.6.2 False negatives

Conversely, certain cheating behaviors remain undetected, particularly when they involve subtle or minimal motion. Examples include brief eye movements, discreet glances, or low-amplitude gestures that do not produce strong visual deviations. These cases are especially challenging when the anomaly is temporally short or partially occluded, limiting the model's ability to distinguish them from normal activity.

#### 6.6.3 Temporal limitations

A key limitation arises from the temporal structure of anomalies. In the Cheatomaly dataset, cheating behaviors are typically sparse and short relative to the full video duration. Within the Multiple Instance Learning framework, anomaly scores are aggregated at the video level, which can dilute the contribution of short anomalous segments when surrounded by long normal sequences. This affects both ranking performance and temporal localization.

#### 6.6.4 Contextual ambiguity

More fundamentally, cheating behaviors are highly context-dependent. Actions such as looking sideways, adjusting posture, or interacting with objects may be either normal or suspicious depending on situational context that is not fully captured by visual features alone. This ambiguity limits the effectiveness of purely visual representations and highlights the difficulty of modeling such behaviors without additional contextual information.

#### 6.6.5 Discussion

These observations indicate that the main limitations of the proposed approach are not solely due to the model architecture, but are strongly tied to the intrinsic properties of the task. The combination of subtle motion, temporal sparsity, and contextual ambiguity makes cheating detection particularly challenging under weak supervision. As a result, errors arise both from visual similarity between normal and abnormal behaviors and from the limitations of ranking-based aggregation in capturing short-duration anomalies.

### 6.7 Discussion

The experimental results demonstrate that the proposed weakly supervised anomaly ranking framework achieves strong video-level performance while providing meaningful temporal localization, despite the absence of frame-level supervision. This confirms that ranking-based formulations are well-suited for scenarios where precise temporal annotations are difficult or impractical to obtain.

However, the gap between video-level and frame-level performance highlights a fundamental limitation of the task. Cheating behaviors in real classroom environments are subtle, temporally sparse, and often context-dependent, making precise temporal localization significantly more challenging than global video-level discrimination.

Importantly, both the baseline comparison and statistical analysis show that differences between feature aggregation strategies (Mean vs. MSD) are not statistically significant. This indicates that performance is not primarily driven by the choice of temporal aggregation, but rather by the overall formulation of the problem and the characteristics of the data. In this setting, aggregation strategies mainly influence the balance between ranking stability and temporal sensitivity, without providing consistent gains.

The qualitative analysis further reveals that errors are largely caused by the intrinsic ambiguity of the task, including visual similarity between normal and suspicious behaviors, short anomaly duration, and contextual dependencies that are not fully captured by visual features alone. These limitations suggest that purely visual and weakly supervised approaches may be insufficient to fully resolve such subtle behavioral patterns.

From a broader perspective, the Cheatomaly dataset introduces a challenging and realistic benchmark that differs from traditional anomaly detection datasets. Instead of relying on visually obvious anomalies, it focuses on context-dependent behaviors where deviations from normality are subtle and temporally localized. This shifts the focus from detecting extreme events to modeling nuanced behavioral patterns.

Overall, this work highlights that progress in exam cheating detection is less constrained by model architecture and more by the inherent complexity of the task. Future work may benefit from incorporating richer contextual information, multimodal signals, or stronger temporal modeling to better capture the fine-grained dynamics of cheating behaviors.

## 7 Conclusion

This work addresses the challenge of detecting cheating behaviors in realistic classroom examination environments, where anomalies are subtle, temporally sparse, and strongly context-dependent. To reflect these characteristics, we introduce Cheatomaly, a dataset designed specifically for anomaly ranking in exam scenarios, and formulate the task as a weakly supervised ranking problem rather than a strict binary classification task.

The results demonstrate that this formulation enables effective video-level prioritization of suspicious behaviors while maintaining meaningful temporal localization, despite the absence of frame-level supervision. At the same time, the observed gap between video-level and frame-level performance highlights the inherent difficulty of fine-grained anomaly detection in such settings.

Importantly, both the baseline comparison and statistical analysis show that variations in temporal feature aggregation do not lead to statistically significant improvements. This indicates that performance is primarily driven by the problem formulation and the nature of the data, rather than by specific architectural choices. In this context, modeling subtle and context-dependent behaviors remains the central challenge.

More broadly, this work shifts the focus of anomaly detection in educational environments from detecting visually obvious events to understanding nuanced behavioral patterns. This perspective emphasizes the need for approaches that go beyond purely visual signals and incorporate richer contextual reasoning.

Future work will explore integrating higher-level semantic understanding, particularly through Vision-Language Models, to better capture contextual cues and reduce ambiguity in decision-making. Additionally, extending the dataset across more diverse environments and conditions will be essential to evaluate robustness and generalization in real-world deployment scenarios.

## Data Availability

The dataset used in this study is curated from publicly available online sources. The processed dataset and annotations are available from the corresponding author upon reasonable request. Requests to access the datasets should be directed to Alaoui Mrani El Mehdi, elmehdi.alaouimrani@usmba.ac.ma.
